# New Epidermal-Growth-Factor-Related Insights Into the Pathogenesis of Multiple Sclerosis: Is It Also Epistemology?

**DOI:** 10.3389/fneur.2021.754270

**Published:** 2021-11-26

**Authors:** Giuseppe Scalabrino

**Affiliations:** Department of Biomedical Sciences for Health, University of Milan, Milan, Italy

**Keywords:** epidermal growth factor (EGF), multiple sclerosis pathogenesis, remyelination failure, experimental CNS demyelinating diseases, epistemology

## Abstract

Recent findings showing that epidermal growth factor (EGF) is significantly decreased in the cerebrospinal fluid (CSF) and spinal cord (SC) of living or deceased multiple sclerosis (MS) patients, and that its repeated administration to rodents with chemically- or virally-induced demyelination of the central nervous system (CNS) or experimental allergic encephalomyelitis (EAE) prevents demyelination and inflammatory reactions in the CNS, have led to a critical reassessment of the MS pathogenesis, partly because EGF is considered to have little or no role in immunology. EGF is the only myelinotrophic factor that has been tested in the CSF and spinal cord of MS patients, and it has been shown there is a good correspondence between liquid and tissue levels. This review: (a) briefly summarises the positive EGF effects on neural stem cells, oligodendrocyte cell lineage, and astrocytes in order to explain, at least in part, the biological basis of the myelin loss and remyelination failure in MS; and (b) after a short analysis of the evolution of the principle of cause-effect in the history of Western philosophy, highlights the lack of any experimental immune-, toxin-, or virus-mediated model that precisely reproduces the histopathological features and “clinical” symptoms of MS, thus underlining the inapplicability of Claude Bernard's crucial sequence of “observation, hypothesis, and hypothesis testing.” This is followed by a discussion of most of the putative non-immunologically-linked points of MS pathogenesis (abnormalities in myelinotrophic factor CSF levels, oligodendrocytes (ODCs), astrocytes, extracellular matrix, and epigenetics) on the basis of Popper's falsification principle, and the suggestion that autoimmunity and phologosis reactions (surely the most devasting consequences of the disease) are probably the last links in a chain of events that trigger the reactions. As it is likely that there is a lack of other myelinotrophic growth factors because myelinogenesis is controlled by various CNS and extra-CNS growth factors and other molecules within and outside ODCs, further studies are needed to investigate the role of non-immunological molecules at the time of the onset of the disease. In the words of Galilei, the human mind should be prepared to understand what nature has created.

## Introduction

Epidermal growth factor (EGF) was first isolated from mouse submaxillary glands in 1962 (1), but it was not until 20 years later that it was identified in human cerebrospinal fluid (CSF) (2), and then in rat brain (3). This delay may explain why research into the role of EGF in the central nervous system (CNS) and various experimental and/or human CNS diseases began after investigations of its role in other targets, and the picture is further complicated by the fact that EGF is also produced outside the CNS, mainly by the distal tubles of the kidneys and the enteric nervous system (4).

Our interest in the role of EGF in the CNS began when we found that it is the physiological mediator of cobalamin (Cbl)-induced CNS myelinotrophism as Cbl positively regulates CNS EGF synthesis and CSF EGF levels in the rat (5). We subsequently verified this regulation in the serum and CSF of patients with clinically confirmed severe Cbl deficiency (i.e., those with pernicious anaemia and/or subacute combined degeneration) (5), and then investigated the role of EGF in multiple sclerosis (MS) because subacute combined degeneration is still considered in the differential diagnosis of early MS (6–8); and some cases of late-onset Cbl*C* disease [a genetic defect of Cbl metabolism with a CNS histopathological picture that is similar to that of subacute combined degeneration (5)] have been described as resembling MS (9). We found that Cbl regulation of EGF levels is lost in MS CNS and that EGF is lacking in MS CNS (see further on). Unlike MS, subacute combined degeneration is a pure myelinolytic CNS disease and does not have any of the histopathological or ultrastructural features of demyelination and/or remyelination (5), and there are no changes in CNS neurolipid composition (5), as it instead occurs in MS (10).

The aim of this review is to recapitulate the main positive effects of EGF on the main CNS cells involved directly or indirectly in myelinogenesis; and its role in the pathogenesis of various autoimmune or non-autoimmune-mediated experimental “models” of MS, MS itself, and CNS remyelination failure in MS, as it is demonstrated by the beneficial effects of its *in vivo* administration in some experimental models (see further on). In addition, an outline of the evolution of the concept of cause-effect principle in Western philosophy will be used to discuss epistemologically whether the autoimmune and phlogistic reactions of MS are causes or consequences of the disease. Finally, some not immunology-linked MS abnormalities will be discussed to tackle the problem of the MS pathogenesis from different angles.

## The EGF Role in the Genesis and Maintenance of CNS Myelin

The effects of EGF on neural stem cells (NSCs), oligodendrocytes (ODCs), astrocytes (ASTs), neurons (NEUs), and microglia have recently been reviewed (11), and can be summarised as follows. For further details, the reader is referred to this broader review.

The crucial effect of EGF on the proliferation and differentiation of NSCs was discovered in the 1990s (12, 13). NSCs come from and are distributed in different regions of human, mouse, and rat CNSs [the sub-ventricular zone, the lining of the lateral ventricle, the sub-granular zone of the dentate gyrus within hippocampus, sub-cortical white matter, the sub-callosal zone between the corpus callosum and dorsal hippocampus, and the spinal cord (SC)], act as a reservoir of multipotent cells, and can be expanded by suitable stimuli during and after CNS demyelinating diseases induced in experimental animals by various means (14, 15). They play a major role in relation to CNS demyelinating insults as they can be committed to a neuronal or glial cell lineage by EGF and other neurotrophic growth factors (16); and they can be mobilised *in vivo* from the subventricular zone to demyelinated CNS areas (17–19). Given that most of these factors are also secreted by ASTs, this clearly means that ASTs can function as mediators of CNS myelination by promoting proliferation, differentiation, and migration of ODC precursors (OPCs) (20).

EGF prevents the delay in ODC maturation, generates a great number of new ODCs, and inhibits signalling pathways that stop maturation of OPCs, and anti-EGF antibodies greatly reduce *in vitro* OPC migration and silence EGF receptor activation (21).

EGF induces 2',3'-cyclic nucleotide 3'-phosphodiesterase (EC 3.1.4.37) activity, which is considered to be an ODC marker at all stages of myelination (22). EGF is therefore one of the most powerful and multifarious epigenetic chemical messengers determining the fate and maturation of NSCs, ASTs, and OPCs/ODCs, although other CNS-trophic growth factors, such as platelet-derived growth factor (PDGF), insulin growth factor (IGF), fibroblast growth factor (FGF), transforming growth factor-β, and ciliary neurotrophic factor, also play a role in the differentiation and proliferation of the different CNS cell lineages (20, 23–25).

The EGF induction of the activity of glutamine synthetase (GS, EC 6.3.1.2) merits particular attention, because ASTs are key cells in the maintenance of glutamatergic neurotransmission and indirectly involved in CNS myelinogenesis (26, 27). The glutamate released by glutamatergic NEUs is taken up by ASTs through specific transporters that convert it into glutamine by means of GS; and the glutamine released by ASTs is taken up by NEUs and eventually reconverted to glutamate by means of phosphate-activated glutaminase (26). Glutamate has been shown to regulate the proliferation of OPCs, to instruct them to differentiate into myelinating ODCs, and to induce myelin formation after its release by axons (27), which makes glutamate signalling a powerfully mechanism allowing NEUs and ASTs to regulate the development of OPCs.

EGF is only one tile in the intricate mosaic of CNS myelination, even though it plays a key role by positively regulating the proliferation and differentiation of NSCs toward neuroglial lineages and stimulating the CNS synthesis of the normal cellular prions (PrP^c^s) that are also produced by ODCs (28), and are widely acknowledged to be involved in the genesis and maintenance of CNS myelin (28), and guide the differentiation of human NSCs into committed NEU, ODC, and AST lineages (29). EGF directly stimulates the AST synthesis of IGF (30), which is involved in the positive regulation of OPC differentiation (31), is indispensable for mature ODC survival (32), and induces the differentiation of multipotent adult neuronal progenitor cells into ODCs (33), and modulates OPC Akt activity during the terminal phase of differentiation (34).

EGF is also a positive regulator of thyroid cell proliferation (35), and tri-iodothyronine plays a key role in the timing of OPC differentiation by stopping OPC proliferation and starting their terminal differentiation (36, 37). Therefore, it is no accident that tri-iodothyronine has been successfully used to treat experimental allergic encephalomyelitis (EAE) (36), cuprizone-induced CNS demyelination, and MS (38, 39).

It is interesting to note that Akt is a common step in the signal pathways of EGF, Cbl, IGF-1, Notch, neuregulins (NRGs) [reviewed in (11)], nuclear factor of activated B cells (40), and the mammalian target of rapamycin (41, 42). Overexpression of Akt, which activates mammalian target of rapamycin, results in hypermyelination as a result of an increased number of myelin wraps (43).

Notch signalling is expressed in OPCs, ODCs, and ASTs, and its intracellular domains cross-talks with EGF [reviewed in (11)]. Notch1 has been shown to inhibit *in vitro* OPC differentiation and myelin formation (44). Notch regulates some of the ODC transcription factors that influence the myelin gene expression of ODCs (45). Notch signalling is a central pathway used by ASTs to regulate both the specification of AST precursors from NSCs and their subsequent maturation (46). The EGF-Notch interaction occurs in the subventricular zone and regulates the size of OPC pool (47). Furthermore, AST-derived Endothelin-1 inhibits OPC differentiation and remyelination through Notch activation and Jagged induction in reactive ASTs of a lysolecithin-induced demyelination in mouse CNS (48).

CNS myelin status depends on the balance between myelin-building and myelin-destroying factors located within ODCs, in myelin itself, and in the extra-cellular matrix (ECM) (42, 49, 50). Furthermore, physiological and repair myelination in the CNS require the right expression and combination of various CNS growth factors and membrane-bound cell molecules, particularly integrins (50, 51). EGF increases NSC membrane levels of the β_1_-integrin involved in axonal-glial interactions, Akt-dependent myelin wrapping, and cross-talk with the Notch pathway, and control axonal ensheathment by ODCs (52).

## EGF in Experimental Autoimmune-Mediated or Non-Immune-Induced CNS Demyelinating Diseases and MS

MS is widely considered to be the paradigm of human, acquired demyelinating diseases of the CNS white matter, although the grey matter of the brain and SC are also affected. Grey matter lesions differ, at least in part, from those of white matter, and grey matter damage becomes increasingly dominant as MS progresses (53, 54).

The various experimental MS-like models (EAE, chemically- or virally-induced CNS demyelination, and transgenic animals) do not entirely reproduce the histopathological features, clinical course, or abnormalities in cerebrospinal fluid (CSF) typical of MS, but only mirror some of its characteristics (55–58). This has raised substantial doubts as to whether the experimental and human diseases have the same pathogenesis (59).

It has been found that chronic *in vivo* EGF treatment induces CNS remyelination in lysolecithin- or cuprizone-induced CNS demyelination (19, 60). It has been shown that NRGs [polypeptides sharing an EGF-like signalling domain and belonging to the EGF superfamily and produced by glial cells (61, 62)] are effective in enhancing SC remyelination in mice with myelin basic protein (MBP)-induced EAE or lysolecithin-lysophosphatidyl-choline-induced demyelination, and promoting the local maturation of new OPCs into ODCs (63, 64). Tissue-type plasminogen activator (which is widely expressed in the CNS and whose structural domain is homologous to that of EGF) enhances OPC migration in the corpus callosum after lysolecithin-induced white matter lesions (65, 66). A mouse strain with genetically determined high CNS EGF expression repairs CNS demyelination better than a mouse strain with genetically determined low CNS EGF expression after receiving Theiler's murine encephalomyelitis virus (67). We found that chronic EGF treatment during the development of myelin ODC glycoprotein (MOG)-induced EAE prevents the onset of the disease and strongly reduces the phlogistic reaction, and its “therapeutic” results are even better than those observed in MOG-induced EAE mice chronically treated with dexamethasone (68). A conditioned medium derived from cultured NSCs and ODCs and containing NRG, ciliary neurotrophic factor, and brain-derived neurotrophic factor increases remyelination and decreases inflammation when intranasally administered to mice with MOG-induced EAE (69).

Summarising the findings of the above experimental results, it should be noted that EGF has been shown: (a) to be an effective “cure” for three different experimental models of MS (i.e., the chemically- or virally-induced CNS demyelination, and the EAE); and (b) to prevent and/or “cure” the demyelination induced in different CNS areas (i.e., corpus callosum and SC) of these MS-like models.

NRG levels are markedly decreased in active MS lesions (70), but a previous study showed that *post-mortem* CSF NRG levels are unchanged in MS patients (71). Heparin-Binding-EGF is over-expressed in the ASTs of active lesions (72). We found that EGF levels are decreased in the CSF and serum of MS patients regardless of the course of the disease (11). Furthermore, the positive regulation of EGF levels by Cbl is lost in the CSF of our MS patients, who have lower EGF levels but higher Cbl levels than controls with other neurological diseases. It has also been reported that plasma EGF levels are lower in patients with progressive disease than in those whose disease has a relapsing-remitting course (73). The blood EGF levels of MS patients increase significantly after there has been an improvement in their nutritional and clinical status (74).

We found that the levels of EGF, Cbl, and PrP^c^s in *post-mortem* SC samples of MS patients are significantly lower than in *post-mortem* SC samples taken from patients with other neurological or non-neurological diseases, and the negative Cbl control of PrP^c^ levels in the normal CSF is lost in MS SC, in which the levels of both the molecules are markedly decreased (11). Any remyelination is doomed to failure if and when these three key molecules for whatever type of CNS myelination are lacking. It must however be emphasised that mechanisms underlying the failure of remyelination in MS lesions include scarcity of OPC pool, limited migration of OPCs, the pathological environment (i.e., ECM), loss of axonal signals that support ODC-derived remyelination, and the presence of remyelination inhibitors in ECM and myelin itself (see below).

It is worthwhile recalling here that EGF has been shown to increase myelination in aggregating brain cell cultures and to induce ODC process formation after different types of injury [reviewed in (11)]. However, although EGF abnormalities in the CNS of MS patients play a role in the pathogenesis of the demyelination and/or remyelination failure, they are not disease-specific as they have also been observed in the CNS of patients with amyotrophic lateral sclerosis and in the CSF of schizophrenic patients (75, 76).

Given that EGF does not seem to have any immunological role (77), the findings of studies of EGF in experimental MS-like models (i.e., those in which it has been administered) and in MS itself support the view that the autoimmune reaction in the SC of EAE mice may actually be caused by damage to or abnormalities in the structure of CNS myelin and/or an ODC disease rather than the other way round as is traditionally believed. It is conceivable that the beneficial effects of EGF treatment may be also due to the EGF-induced triiodothyronine secretion (see above) and/or EGF-induced prolactin levels, as demonstrated by the MS remission during pregnancy (78). However, one paper has described the anti-inflammatory effect of Heparin-Binding-EGF *in vitro* (79), and another study has found that NRG treatment increases the CNS levels of interleukin-4 and interleukin-10 during the remyelination phase of MBP-induced EAE (63). PDGF also modifies the immune response of cultured T cells (80).

Some myelin- and/or ODC-trophic growth factors such as IGF, FGF, and PDGF are over-expressed in rodents with experimentally induced CNS demyelinating diseases (81–83), but the expression of myelinotrophic growth factors during and/or after CNS demyelination (however induced) differs from that observed during CNS developmental myelination (84).

## The Epistemology of the Pathogenesis of MS

### The Cause-Effect Relationship in Medicine

The development of any treatment that prevents injury or repairs the axonal/glial interface in MS is a lofty aim, but one that is necessarily doomed to failure if the cause of the disease is unknown. Although it is beyond the scope of this review to discuss in detail how the concept of the relationship between cause and effect developed in Western philosophy, but it is important to do so briefly before applying it to the MS pathogenesis.

At his conference of 1932 entitled “Science and Philosophy,” Alfred Whitehead said that “science and philosophy are different aspects of the same exercise of the human mind” and, as medicine is clearly an experimental science in the philosophic sense of the word, it may be fruitful to consider the MS pathogenesis epistemologically. In a letter to Federico Cesi written on 30 June 1612, Galileo Galilei said “We must not think that nature should conform to what might seem to us to be better and neater set-up, but it behoves us to prepare our minds to understand what nature has created because we can be sure that it is the best, and nothing else.” Galilei was not a philosopher in the modern sense of the word, but he is fundamental to the history of Western philosophy, because he established the new methodology and type of knowledge that we call science. Although he was essentially referring to physics, his statement was transposed to medicine by Claude Bernard who in his “Introduction to the study of the experimental medicine,” wrote that “There are two types of experimenter: some sacrifice the facts to their ideas, whereas others sacrifice their ideas to the facts found in their research” and that his intention was “To demonstrate that biology cannot have a different basis from that of the other sciences and so there cannot be any difference between the tenets of the biological sciences and those of the chemical and physical sciences” (85), although this can be done with some limitations (86). He also distinguished “observers” from “experimenters,” because the former merely look for a biological phenomenon, whereas the latter look for a phenomenon modified in accordance with their hypothesis (85). Bernard's merit is not that of inventing the experimental method of science, but that of applying the known method of physics and chemistry to medicine. It is not casual that Henri Louis Bergson defined the Bernard's “Introduction” as the Cartesian “Discourse on the Method” of the nineteenth century (87).

It is well-known that British philosophy traditionally criticised the principle of cause-effect from the time of William of Ockham, this reached its acme with the radical denial of John Locke (who was a physician before he was a philosopher) in “An Essay concerning Humane Understanding” (88) and David Hume in “A Treatise of Human Nature: Being an Attempt to introduce the experimental Method of Reasoning into Moral Subjects,” in which he wrote that “Objects have no discoverable connexion together; nor is it from any other principle but custom operating upon the imagination, that we can draw any inference from the appearance of one to the existence of another” (89). John Stuart Mill also belonged to the British tradition of “empirical gnoseology” insofar as he agreed with Hume in denying the principle of cause-effect, and thought it was useless to try to justify it because of the mental association between each person and his/her experience (90). He wrote that “all inference is from particulars to particulars” (90).

In his book “Of the seats and causes of diseases as investigated by means of the anatomical method” (91), Giovanni Battista Morgagni (the founder of modern pathology) correlated the symptoms of patients with the pathological lesions observed in their organs at the time of autopsy. In Morgagni's time, the word “morbus” (disease) was used to described symptoms, and it was thought that their cause(s) could be localised by identifying the most morphologically affected organ(s). However, when the word “disease” subsequently took on its current meaning of an abnormal physiopathological condition, whose symptoms are simply epiphenomena, it was realised that the histopathological lesions of a diseased organ are the consequence and not the cause of a “disease,” and its cause(s) are to be found inside the body (e.g., abnormal genotypes, epigenetic changes, altered homeostatic mechanisms, cellular pathologies, and developmental defects) and/or outside it (e.g., viruses, bacteria, parasites, and radiations).

The second half of the nineteenth century saw a revival of the principle of cause-effect in medicine as a result of the discoveries of *Bacillus anthracis* (1876) (92) and *Mycobacterium tuberculosis* (1882) by Robert Koch (93). In the same time, Louis Pasteur succeeded in culturing the causative agent of fowl cholera (94) and then proposed the “germ theory” to explain all infectious diseases (95). This led to the idea that each disease had a precise aetiology and that the penetration of a body by a microbe necessarily gave rise to a specific disease. Koch also formulated his three postulates of isolating bacilli from people and animals with the disease, growing the bacilli in culture, and reproducing the disease by inoculating the bacilli in animals (96).

In his “Determinism and Indeterminism in Modern Physics” (97), Ernst Cassirer wrote that “the transcendent [in the Kantian sense of the word (writer's note)] analysis of the concept of causality, which has been opposed by Immanuel Kant in his “Critique of Pure Reason” (2nd edition, 1787) to the psychological analysis of Hume, is not dealing with the being of the things and their mutual connexion, but rather only with the way through which we know the things, the form of the objective knowledge.”

The beginning of epidemiological studies during the last century led to a greater understanding of causality (more than a single factor can lead to a given outcome) and an increasing awareness of the importance of possible concauses in the pathogenesis of a given disease. In “The Logic of Scientific Discovery” (98), Karl Popper introduced the falsificationist method of science, which requires that any scientific hypothesis must be disproved. In other words, if we want a theoretical system to be controlled by experience, the demarcation criterion is not its verification but its falsifiability. Popper wrote in “Conjectures and Refutations” that “methodological falsification should be the bedrock of science and human thought in general (99), because a critical stance and the tradition of freely discussing theories in order to identify their weak points and thus improve them is the most reasonable and rational position.”

In “Against method. Outline of an anarchistic theory of knowledge” (100), Paul Feyerabend wrote: “A complex medium containing surprising and unforeseen developments demands complex procedures and defies analysis on the basis of rules that have been set up in advance and without regard to the ever-changing conditions of history” and “The history of science, after all, does not consist of facts and conclusions drawn from the facts. It also contains ideas, interpretations of facts, problems created by conflicting interpretations, mistakes, and so on.” According to him, modern philosophy has given too much attention to understanding scientific practise rather than concentrating on scientific method, and so it is time for anarchism to replace rationalism in the theory of knowledge.

In much of the literature, MS is defined as a chronic autoimmune CNS demyelinating disease with a phlogistic reaction due to a failure to distinguish self-antigens (myelin) from foreign antigens, thus indicating that it is one of the primary human autoimmune diseases and that, by definition, its cause is autoimmunity; however, other authors define it as a chronic autoimmune CNS demyelinating disease of unknown cause. The difference is not merely semantic because the first definition simply seems to be inexact and even misleading, whereas the second gives more accurate information insofar as it recognises the existence of an autoimmune reaction and acknowledges the fact that we still do not know real cause of the disease.

It is no accident that Bruce Trapp and Klaus-Armin Nave entitled their review “Multiple sclerosis: An immune or neurodegenerative disorder?” (101) and Peter Stys entitled his reviews “Multiple sclerosis: autoimmune disease or autoimmune reaction?” (102) and “Pathoetiology of multiple sclerosis: are we barking up the wrong tree?” (103).

It is widely accepted that the traditional autoimmune MS pathogenesis is not definite and beyond any reasonable doubt, and even denied by various authors (101–107). Using Koch's postulates (96) as a means of understanding the MS pathogenesis is hampered by its surely multi-factorial nature and the fact there is no suitable experimental model of the disease (55, 56, 58, 107); for example, the viral hypothesis seems to be weakly grounded because none of the many putative viruses has been unequivocally proved to be responsible for the disease (6, 8, 58, 108). The question is further complicated by the fact that MS is probably not one disease, given the differences in its clinical course and histopathological features, and the degree of remyelination varies greatly during the course of the disease and in different parts of the CNS (109, 110). MS pathogenesis is the sum of several factors where the consequences may be more harmful than the causes and where demyelination/remyelination steps are points of crucial interest in the patient's recovery. Even genetically, histopathologically, and proteomically MS is a heterogeneous disease (106, 111).

The best way of identifying the MS cause(s) currently seems to be to extend investigations of changes in the different molecules in patients' CSF and/or CNS samples (112). This approach has of course already been used for a long time, but it needs to be broadened in order to be able to collect the large amount of information that would make it possible to put together the pieces of a mosaic that would explain what triggers the autoimmune response and phlogistic reaction.

The different models of EAE and chemically- or virally-induced CNS demyelinating diseases have the drawbacks that investigators know the cause of the induced diseases *a priori* and, unfortunately, the models do not reproduce the MS reality (see above). In other words, the temporal sequence (the injection of a myelin antigen, a chemical toxin or a virus and the subsequent onset of MS-like CNS lesions) is well-established and only apparently reproduces a hypothetical cause-effect relationship for MS.

Some of these models seem to be examples of the famous Latin tag “*post-hoc, ergo propter hoc*” (after this, therefore because of this), but this may also be a sophism because, although the causes of these models are clearly known, this does not necessarily mean that they are the cause(s) of MS. Proof that a given condition always precedes an observed phenomenon in clinical medicine does not mandatorily mean that it can be definitely inferred to be the direct cause; it must also be demonstrated that the phenomenon is not observed when the condition is removed or, in the words of Claude Bernard (85), “sublata causa, tollitur effectus” (upon removal of the cause, the effect is removed).

The classical three steps of experimental medicine according to Claude Bernard [observation, hypothesis, and experimental testing of the hypothesis (85)] cannot be followed when investigating the MS pathogeneris in the absence of true animal models, and the same goes for any research into the mechanism(s) underlying the axonal loss that is the final common pathway of the disease (113–115). Impulse conduction along demyelinated axons requires considerably more energy than along myelinated axons, but inflammation, demyelination, and reduced mitochondrial gene expression in MS contribute to energy failure (115–117).

## Is There Enough Evidence to Review the Pathogenesis of MS In Terms of A Primary Abnormality In ODCS and/or CNS Myelinotrophic Growth Factors?

The aporias of the autoimmune pathogenesis of MS have been thoroughly discussed in a number of reviews (101–107). It is clearly very important to identify what triggers the autoimmune and phlogistic reactions. Six main points supporting a non-autoimmune MS pathogenesis must be briefly discussed: abnormalities in CNS-trophic growth factor expression; CNS glutamate excitotoxicity; ODC abnormalities; myelin and GFAP abnormalities; ECM abnormalities; and epigenetic abnormalities.

### CNS-Trophic Growth Factor Abnormalities

It is widely recognised that CSF abnormalities very often mirror abnormalities in CNS tissue and therefore CSF analyses are very important (112, 118). There has been a long-running debate about the role of neurotrophic growth factors in EAE and chemically- or virally-induced CNS demyelinating models (see above), but the same cannot be said about MS (i.e., CSF and CNS samples from MS patients).

Other than EGF (see above), PDGF and FGF have been assayed in the CSF of MS patients, and it has been found that high PDGF levels are associated with prolonged relapse-free survival (119); that patients with a primary progressive disease course have significantly reduced PDGF levels (120); that PDGF levels decrease during the course of progressive disease, thus indicating that patients with prolonged MS are unable to produce PDGF to repair damaged myelin (121); and that PDGF levels are significantly higher in patients whose symptoms have completely recovered than in those who show incomplete symptom recovery after a relapse (120). This PDGF deficiency seems to be important because it has been shown that PDGF improves the density and proliferation of ODCs during demyelination, promotes the remyelination of chronically demyelinated lesions, and acts as a mitogenic factor for NSCs and adult OPCs (122, 123). However, only EGF has been assayed in both CSF and SC of MS patients.

CSF FGF levels are significantly higher in MS patients regardless of the clinical course of the disease (even in those with clinically isolated syndrome) than in controls, and may contribute to disease progression (124). Furthermore, exogenous FGF down-regulates GS activity (125), and so a possible simultaneous presence of excess FGF and EGF deficiency in MS patients may simultaneously further decrease this activity, thus impairing glutamate homeostasis and consequently worsening NEU degeneration and causing ODC demise (see also below). Furthermore, it has been shown that the MS CSF contains antibodies against an OPC-specific surface glycoprotein (AN2) that is likely to contribute to blocking remyelination (126).

The analyses of CSF growth factor levels indicate the presence of a derangement of those that are pivotal for the maturation of the ODC lineage and the formation of myelin (i.e., EGF and PDGF), but have so far not been found to play any clear role in immunology. It is conceivable that CSF growth factor derangement may lead to abnormalities in OPC- ODC lineage or that OPC/ODC abnormalities lead to inappropriate growth factor production, thus triggering a vicious circle.

### Glutamate Excitotoxicity

Tight regulation of excitatory aminoacids such as glutamate (mediated by N-methyl-D-aspartate receptors and α-amino-3-hydroxy-5-methyl-4-isoxazolepropiomic acid /kainate receptors) should be maintained in the CNS, because its accumulation causes harmful NEU excitation and glial activation, that eventually leads to the death of particularly ODCs (127, 128). ODCs are vulnerable to an increase in extracellular glutamate because they express α-amino-3-hydroxy-5-methyl-4-isoxazolepropionic acid- and N-methyl-D-aspartate-receptors (26, 128). Glutamate transporters are present in ODCs, but they are expressed at higher levels in white matter ASTs than those observed in grey matter ASTs (129), another way by which ASTs contribute to maintaining ODC viability and myelin integrity (128, 130).

Interestingly, it has been found that the lesions of active and silent MS lack GS (130), and this contributes to increased glutamate excitotoxicity in MS because glutamine synthesis by GS is a means of preventing the increase in extra-cellular glutamate and thus detoxifying the excess glutamate in ASTs (26). This may indicate that the excitotoxicity induced by glutamate excess is largely independent of autoimmune and/or phlogistic reactions, although it has been suggested that the increased glutamate levels in MS CNS may be also due to over-production by inflammatory cells and microglia (127, 131). Nonetheless, it remains to be elucidated whether increased extracellular glutamate concentration is accumulated in MS extracellular space by an ineffective glutamate cell uptake or by an abnormal release of this neurotransmitter from NEUs and/or non-neuronal cell or by both these concurrent abnormalities (127). CSF glutamate levels are higher during the relapse phase than in controls and patients with relapsing-remitting disease examined during a stable clinical phase (132, 133).

### OPC/ODC Abnormalities

It must first be emphasised that adult OPCs are different from perinatal OPCs: they migrate more slowly, have a different cell cycle time, and show different responses to growth factors that are partly due to their transcriptome profiles (134). They also generate much shorter but many more internodes than ODCs during post-natal myelination (135–138). In response to demyelination, adult OPC undergo a switch to a state that is characterised by increased expression of various transcription factors (23, 134).

Various lines of evidence support the idea that MS patients are highly likely to develop a primary oligodendrocytopathy. Autopsy findings indicate that Notch signalling is activated in the brain OPCs of MS patients, although distinct types of MS lesions may express Notch differently (139); the nuclear translocation of the Notch intracellular domain, which is required for myelinogenesis, is missing in MS OPCs (139); and Notch receptors are activated in the brain ODCs of MS patients (140). Furthermore, substantial ODC death has been observed in MS lesions and triggers an autoimmune response against CNS myelin in a mouse model (106, 141); this suggests that something similar may happen in MS (140), although it has been shown that widespread ODC death in conjuction with immune activation does not trigger anti-CNS immunity (142).

The number of OPCs is reduced in chronic MS lesions and they do not mature into ODCs (143). Autopsy findings indicate a deficiency in transcription factor myelin-regulatory-factor-expressing ODCs (crucial for the transition of ODCs from a premyelinating to a myelinating phenotype during remyelination and myelin maintenance (144)) in chronically demyelinated lesions (145). Finally, extensive ODC apoptosis occurs in MS patients (106, 109), and although OPCs are still present in chronic lesions, their significantly smaller number than in early lesions suggests an arrested differentiation (143). It has been shown an activation of gliogenesis in the subventricular zone of *post-mortem* MS brain and this suggests a mobilisation of the subventricular zone-derived glial progenitors toward the lesion zones (146).

The deletion of CNS PrP^c^ exacerbates the autoimmune reaction in EAE mice with a normal immune system (147, 148), and CNS PrP^c^ deficiency delays ODC differentiation in mice (28).

Although there is no doubt that ODCs produce a wide range of immunoregulatory factors and play a role in activating microglia to clean myelin debris (149), it should be better to look even more carefully at the myelinogenic site of ODC coin and the potential NSC development and migration, because this may help us to answer the age-old question of whether ODCs are culprits or victims in the MS pathogenesis, and to build up a potential endogenous remyelinating therapy, possibly grounded on the stimulation of proliferation and migration of NSCs toward the CNS demyelinated areas.

### Myelin and GFAP Abnormalities

The debate on the presence of abnormal myelin in MS begun with the pioneering observations of Moscarello and his colleagues, who found that the Marburg type of fulminating MS is associated with developmentally immature MBP (150, 151). MBP is more citrullinated in MS, and more deiminated but less phosphorylated than MBP in normal white matter (150). Citrullination converts an arginine residue to standard citrulline (151), and this difference in MBP causes a major change in myelin structure, because MBP makes an important contribution to the construction of CNS myelin and the preservation of myelin stability (152). MBP hypercitrullination is due to an increase in the activity of peptidylarginine deiminase (PAD)-2, which is responsible for the deimination of arginyl residues in MBP (151). The promoter region of *PAD2* gene encoding PAD2 is hypomethylated in MS but not in other neurodegenerative diseases, leading to an overexpression of the enzyme (152). It has been shown that the PAD inhibition significantly decreases MBP hypercitrullination in MOG-induced mouse EAE and mitigated the neurological damage in mice with cuprizone-induced CNS demyelination (153, 154). The hypercitrullination of MBP and GFAP has also been found in areas of the CNS that are undergoing demyelination and in the brain and SC of mice with MOG-induced EAE (155). Moreover, MBP hypercitrullination changes its T cell epitope (156).

Exon-2 MBP gene products are expressed at very low levels in the adult CNS, but are increased in MS patients, particularly during the remyelination of chronic lesions (157).

High GFAP levels have been found in MS CSF and were unaffected by relapses, also because MS is characterised by widespread astrocytic activation and astrogliosis (158). Indeed, GFAP has been detected in demyelinated plaques of MS brain (78, 158).

### CNS ECM Abnormalities

It is widely accepted that the ECM molecules, most of which are produced by ASTs (129, 159), affects nearly all aspects of CNS development and functions, regulates the differentiation of the OPC-ODC lineage that ultimately generates mature ODCs (160–162). Therefore, ASTs influence ODCs *via* modification of ECM (129, 159), which plays an important role in providing a healthy environment for CNS myelination (129). Furthermore, ASTs communicate with and influence OPC- and ODC-activity *via* secretion of some ECM components (160). ASTs are connected to other glial cells *via* gap junctions, allowing free flow of ions and small molecules. Gap junctions between ASTs and between ASTs and ODCs are made up of connexins, which are also affected in MS (160). This AST-ODC coupling is essential for myelinogenesis (115).

The ECM of the MS CNS is hostile to remyelination because of its over-expression of some of the inhibitors expressed constitutively or after demyelinating injury or disease (129, 160–163). Furthermore, ECM myelin membrane debris has powerful inhibitory effects on OPC differentiation and ODC myelination (161). The main ECM abnormalities in MS are: decreased tenascin C expression in acute plaques, but increased in chronic plaques; the weak expression of fibronectin (which inhibits ODC differentiation) in shadow plaques, but its aggregates accumulate in response to demyelination; the up-regulation of hyaluronan and its transmembrane binding-protein CD44 in demyelinated lesions, and its down-regulation in partially remyelinated areas; accumulation of the chondroitin sulphate proteoglycans that form a non-permissive substrate for the recruitment and morphological differentiation of OPCs and ODCs and axonal regrowth; excessive myelin debris and/or its insufficient clearance by microglia; the re-expression of the polysialylated form of neural cell adhesion molecule (a negative regulator of CNS myelination) in demyelinated axons; some semaphorins; and the presence of Leucine-rich repeat and Ig domain Nogo receptor-interacting protein, and MOG (159–163). It can therefore be concluded that the degree of remyelination depends on the ratio between OPC differentiation-inhibiting and differentiation-promoting molecules (e.g., laminis) in the ECM (48, 159–163), like that occurs for normal OPC/ODC lineage differentiation (164).

Unfortunately, very little information has been given about the possible effects of EGF on various components of CNS ECM in health and disease. Therefore, there is a strong need for further research in this field, even to achieve a much better understanding of the pathobiology of failed remyelination in MS.

### Epigenetic Abnormalities

Last but not least, it is necessary to mention the epigenomic modifications occurring in MS patients, which involve DNA methylation, histone changes, and non-coding RNAs, and regulate the progression of the ODC lineage and CNS myelination (165–169). For example, the genes responsabile for ODC survival and myelination are hypermethylated and therefore down-regulated in MS brain (170, 171); and DNA methyltransferases are up-regulated in demyelinated areas of the hippocampus of MS patients, whereas normal levels are required for efficient remyelination (169, 170). The epigenetic changes contribute to the MS pathogenesis, and can hardly be reconciled with the theory of autoimmunity. Furthermore, MS brain OPCs and ODCs show transcriptional and epigenomic changes, thus suggesting the possibility of a MS-specific ODC lineage (172, 173).

## Vitamin D and EGF

It is well known that vitamin D deficiency has been proposed as a significant risk factor in MS development (174, 175). The neurotrophic role of vitamin D results from its up-regulation of the synthesis of glial-cell-line-derived neurotrophic factor and neurotrophin 3, and from its down-regulation of neurotrophin 4 (175–177). Moreover, vitamin D enhances NSC proliferation and ODC differentiation (178). Vitamin D administration has been shown to prevent onset of “clinical” symptoms in different EAE models and to block progression of the disease (179, 180). Nevertheless, to the best of my knowledge, there is no any study dealing with the relation between vitamin D and EGF in mammalian CNS.

## Conclusions

On the basis of the mechanisms involved in the MS pathogenesis and failure of remyelination, Robin Franklin proposed a “dysregulation” hypothesis (181), in which remyelination failure reflects an inappropriate sequence of events. The results obtained over the last 20 years and dealing with the different aspects of MS field seem support this hypothesis, although it may be better to call it the “derangement” of key intra-ODC and intra-ECM factors and molecules, because some of them are overexpressed whilst others are lacking. It is therefore of primary importance to address this derangement and migration and proliferation of NSCs, in order to restore a complete process of remyelination (24).

Feyerabend argued that scientific advances can only be understood in a historical context (100). The pioneering studies of Thomas Rivers et al. describing EAE opened up the possibility of inducing a new experimental CNS demyelinating disease (182, 183) but the contribution of this to our understanding of the MS pathogenesis has been limited. However, between the publication of the two reports by Rivers et al. (182) and Rivers and Schwentker (183), Theiler described the viral aetiology of “spontaneous” encephalomyelitis in mice (184), and there is a clear epistemological difference between this and the works of Rivers and colleagues: Theiler described the true cause (i.e., a virus belonging to the picornaviridiae family) of a previous unknown CNS disease, whereas Rivers et al. only described an experimental procedure to induce CNS demyelination as a means of investigating acute disseminated encephalomyelitis (55–57). In keeping with this, there are some differences between EAE and Theiler's encephalomyelitis and, as said above, both are significantly different from MS (185). EAE models have thus contributed to blurring the cause-effect relationship of MS.

Popper pointed out that the methodology of science can be summarised in the three words: “Problems, theories, and criticisms” and that “Each theory must be checked by the facts, and the result of this matching may be double: either confirmation of the theory or its refutation, i.e., its verification or its falsification” (99). Although MS has a broad spectrum of pathological features (see above) and its clinical symptoms do not solely arise from demyelination, chronic demyelination leads to axonal loss (113, 114). MS remyelination necessarily entails the remodelling of some key CNS molecules mentioned above, because newly formed myelin is not the same as that present before demyelination (see above). Moreover, the dysregulation of Wnt-β-catenin signalling in OPCs results in a great delay of both developmental myelination and remyelination (42, 186–188), although there is a report showing the opposite (189). Of note, Wnt pathway is still active in MS lesions (186).

All of these elements seem to be causally independent and unrelated to any autoimmune reaction and therefore MS may start as an oligodendrocytopathy that may also involve ASTs. In other words, the findings observed in MS samples over the last 20 years seem to support the view that autoimmunity and phlogosis are the last (but probably the most CNS-damaging) of a series of events, which suggests that the MS pathogenesis cannot be explained by autoimmunity alone. In science, not seeing what you are looking for does not mean that it does not exist, and one may not be observing the whole context, maybe one is just looking in the wrong direction.

I would like to close with the words of Fragment 18 of Heraclitus:


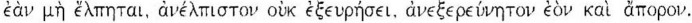


(If you do not expect the unexpected, you will not find it, for it is hard to seek out), because what could be more “unexpected” than the definite determination of the MS pathogenesis?

## Author Contributions

GS declares to be the only author of the review, conceived the topic of the review, collected the papers, and wrote the manuscript.

## Funding

This work was supported by Open access to some Libraries of the Faculty of Medicine and Surgery of the University of Milan to collect most of the papers.

## Conflict of Interest

The author declares that the research was conducted in the absence of any commercial or financial relationships that could be construed as a potential conflict of interest.

## Publisher's Note

All claims expressed in this article are solely those of the authors and do not necessarily represent those of their affiliated organizations, or those of the publisher, the editors and the reviewers. Any product that may be evaluated in this article, or claim that may be made by its manufacturer, is not guaranteed or endorsed by the publisher.

## Abbreviations

AST: astrocyte
Cbl: cobalamin
CNS: central nervous system
CSF: cerebrospinal fluid
EAE: experimental allergic encephalomyelitis
ECM: extracellular matrix
EGF: epidermal growth factor
FGF: fibroblast growth factor
GFAP: glial fibrillary acidic protein
GS: glutamine synthetase
IGF: insulin growth factor
MBP: myelin basic protein
MOG: myelin oligodendrocyte-specific glycoprotein
MS: multiple sclerosis
NEU: neuron
NRG: neuregulin
NSC: neural stem cell
ODC: oligodendrocyte
OPC: oligodendrocyte precursor
PAD: peptidylarginine deiminase
PDGF: platelet-derived growth factor
PrP^c^: normal cellular prion
SC: spinal cord.
